# The significance of serum levels of soluble interleukin-2 receptor in patients undergoing maintenance hemodialysis

**DOI:** 10.1080/0886022X.2020.1761388

**Published:** 2020-05-13

**Authors:** Xiaohong Chen, Yang Li, Xiaoqiang Ding, Jianzhou Zou, Bo Shen, Zhonghua Liu, Wenlv Lv, Xuesen Cao, Fangfang Xiang

**Affiliations:** a Shanghai Institute of Kidney and Dialysis, Shanghai, China; b Shanghai Key Laboratory of Kidney and Blood Purification, Shanghai, China; c Hemodialysis Quality Control Center of Shanghai, Shanghai, China; d Department of Nephrology, Zhongshan Hospital, Fudan University, Shanghai, China; e Shanghai Medical Center of Kidney, Shanghai, China

**Keywords:** Soluble interleukin-2 receptor, hemodialysis, correlation, risk, prognosis

## Abstract

**Background:**

Elevated serum levels of sIL-2R are commonly observed in patients undergoing maintenance hemodialysis (MHD). However, the clinical implications in these subjects are unclear. This study is aimed to assess the significance of elevated sIL-2R levels in MHD patients.

**Methods:**

A total of 382 MHD patients were followed-up from September 2016 to December 2019. Patients were divided into two groups: high sIL-2R, with sIL-2R levels ≥2-fold of the upper limit of normal (710 U/ml); and low sIL-2R, with sIL-2R levels < 2-fold the upper limit of normal. The relationships between sIL-2R levels and other clinical parameters, as well as patient prognosis were both assessed.

**Results:**

The median concentration of sIL-2R was 1268 U/mL. A total of 372 (97.38%) patients exhibited sIL-2R levels higher than the upper limit of the normal range. Multiple linear regression analysis revealed that monocyte count (β = 0.1571, *p* = 0.01), and β_2_-MG (β = 0.2635, *p* < 0.0001), hemoglobin (β = −0.1610, *p* = 0.001), SCr (β = −0.3471, *p* < 0.0001), and HDL-C (β = −0.1091, *p* = 0.029) levels were independent factors influencing serum concentrations of sIL-2R. High sIL-2R was significantly correlated with non-cardiovascular-related mortality (OR 2.97 [95% CI 1.59–5.56; *p* = 0.001), of which 39 (82.98%) were attributed to infection and/or cancer.

**Conclusions:**

Elevated sIL-2R is prevalent in MHD patients and related with several unfavorable parameters. sIL-2R appears to have no ability to predict cardiovascular mortality, which accounts for approximately one-half of all deaths. However, sIL-2R may be beneficial in predicting noncardiovascular mortality.

## Introduction

Interleukin-2 (IL-2), a type of lymphokine mainly produced by T lymphocytes, stimulates an immune response in target cells by binding to the high-affinity IL-2 receptor (IL-2R), which comprises of three subunits (α, β, and γ chains) [1]. Soluble IL-2R (sIL-2R), which was first described by Rubin et al. [2], is a soluble form of the α-chain of IL-2R and spontaneously released from the cell surface by proteolytic cleavage after stimulation. Serum levels of sIL-2R measured in the healthy population are not high, but increase when immune cells are activated by various pathophysiological stimuli. Thus, the release of sIL-2R can be regarded as a characteristic marker of immunocyte activation [2].

Evidence from previous studies has suggested that both uremic status and dialysis therapy may induce high proportions of pre-activated immune cells in the circulation [3–5]. Therefore, elevated serum levels of sIL-2R in patients with end-stage renal disease (ESRD) undergoing maintenance hemodialysis (MHD) has been reported in various studies [6,7]. Increased sIL-2R levels can interfere with regulatory processes because it maintains the ability to bind IL-2 in several other disorders, such as autoimmune, inflammatory and graft-versus-host diseases, and cancer. sIL-2R has been reported to play a functional role in the course of these diseases and be correlated with patient prognosis [8]. However, the significance of elevated sIL-2R levels in patients undergoing MHD is not completely understood. As such, we aimed to evaluate the serum levels of sIL-2R in MHD patients and to determine whether sIL-2R could serve as a prognostic factor for disease prognosis in this patient population.

## Materials and methods

### Study population

ESRD patients in a stable state, free of HIV infection, and undergoing MHD at the Blood Purification Center of Zhongshan Hospital, Fudan University (Shanghai, China) for >3 months were enrolled in this study. Individuals <18 years of age, those with lymphoproliferative disorders, autoimmune disease, history of transplantation, being treated with immunosuppressive agents or IL-2, those with recent infections within 3 months before the study, and those exhibiting evidence of cancer at enrollment were excluded. Patients with a history of cancer treatment but without recurrence or metastasis of their disease before this study were also enrolled. All eligible patients were followed-up prospectively from September 2016 to December 2019.

This study was approved by the Ethics Committee of Zhongshan Hospital, Fudan University (Approval Number: B2013-139). Informed written consent for participation in this study was provided by all participants before enrollment.

### Hemodialysis procedures

Most patients in the blood purification center received hemodialysis (HD) 5 times plus hemodiafiltration (HDF) once every 2 weeks at 4 h per session. In addition, <1/10 of patients underwent HD 3 times plus HDF once every 2 weeks at 5 h per session, depending on their residual renal function. Dialysis sessions were performed by using synthetic dialyzer membranes. The quality of dialysis water conformed to standards described by the Association for the Advancement of Medical Instrumentation and was checked every month.

### Data collection

Demographic information, such as age, sex, and body mass index (BMI), and clinical characteristics including primary kidney disease, vascular access for dialysis, dialysis frequency, dialysis vintage, and single-pooled Kt/V (spKt/V), were collected. spKt/V was calculated based on the reduction in the serum urea concentration during dialysis and calculated using the Daugirdas formula.

Blood samples were obtained at the beginning of this study to test biomedical indexes, including sIL-2R, blood cell counts (neutrophils, lymphocytes, and monocytes), hemoglobin, high-sensitivity C-reactive protein (hs-CRP), albumin, urea, serum creatinine (SCr), uric acid (UA), beta-2-microglobulin (β2-MG), intact parathyroid hormone (iPTH), calcium, phosphorus, ferritin, lipids (total cholesterol [TC], triglycerides [TG], low-density lipoprotein cholesterol [LDL-C], and high-density lipoprotein cholesterol [HDL-C]), homocysteine (Hcy), and NT-probrain natriuretic peptide (NT-proBNP).

### sIL-2R and patient groupings

The normal reference range for serum sIL-2R at the authors’ hospital is 223–710 U/mL, which was determined using a commercially available chemiluminescence enzyme immunoassay (Siemens Healthcare Diagnostics, Shanghai, China) on the IMMULITE 1000 analyzer (Siemens Healthineers Global).

Patients were divided into 2 groups: high sIL-2R, with sIL-2R levels ≥2-fold of the upper limit of normal (710 U/mL); and low sIL-2R, with sIL-2R levels <2-fold the upper limit of normal.

### Statistical analyses

Categorical variables are expressed as number and percentage (%) and compared using chi-squared analysis or Fisher’s exact test, where appropriate. Continuous variables are expressed as mean ± standard deviation (SD) or median with interquartile range (IQR), and compared using the Student’s *t*-test or Wilcoxon rank-sum test. Correlations between sIL-2R and other clinical parameters were examined using Spearman’ s correlation and backward stepwise multiple linear regression analysis. Differences in survival according to sIL-2R levels were analyzed using the Kaplan–Meier method and the log-rank test to detect statistical differences. Risk factors related to mortality were estimated using a logistic regression model and expressed as odds ratio (OR) with corresponding 95% confidence interval (CI). The predictive performance for mortality was evaluated by calculating the area under the receiver operating characteristic curve (ROC [AUC]).

All analyses were performed using STATA version 15.0 (Stata Corporation, College Station, TX, USA). Differences with *p* values < 0.05 were considered to be statistically significant.

## Results

### Patient characteristics

A total of 382 patients undergoing MHD were enrolled in this study, including 234 men (61.26%) and 148 women (38.74%), with a mean age of 60.26 ± 14.00 years (range, 21–88 years) and a median dialysis duration of 4.59 years (range, 0.29–26.68 years) before the study. The etiologies of ESRD were as follows: primary glomerulonephritis (*n* = 200 [52.36%]); diabetes mellitus (*n* = 73 [19.11%]); hypertension (*n* = 38 [9.95%]); polycystic kidney disease (*n* = 36 [9.42%]); obstructive uropathy (e.g. prostatic hyperplasia, urinary stone) (*n* = 19 [4.97%]); tubulointerstitial nephritis (*n* = 7 [1.83%]); and others (*n* = 9 [2.36%]).

**Figure 1.**
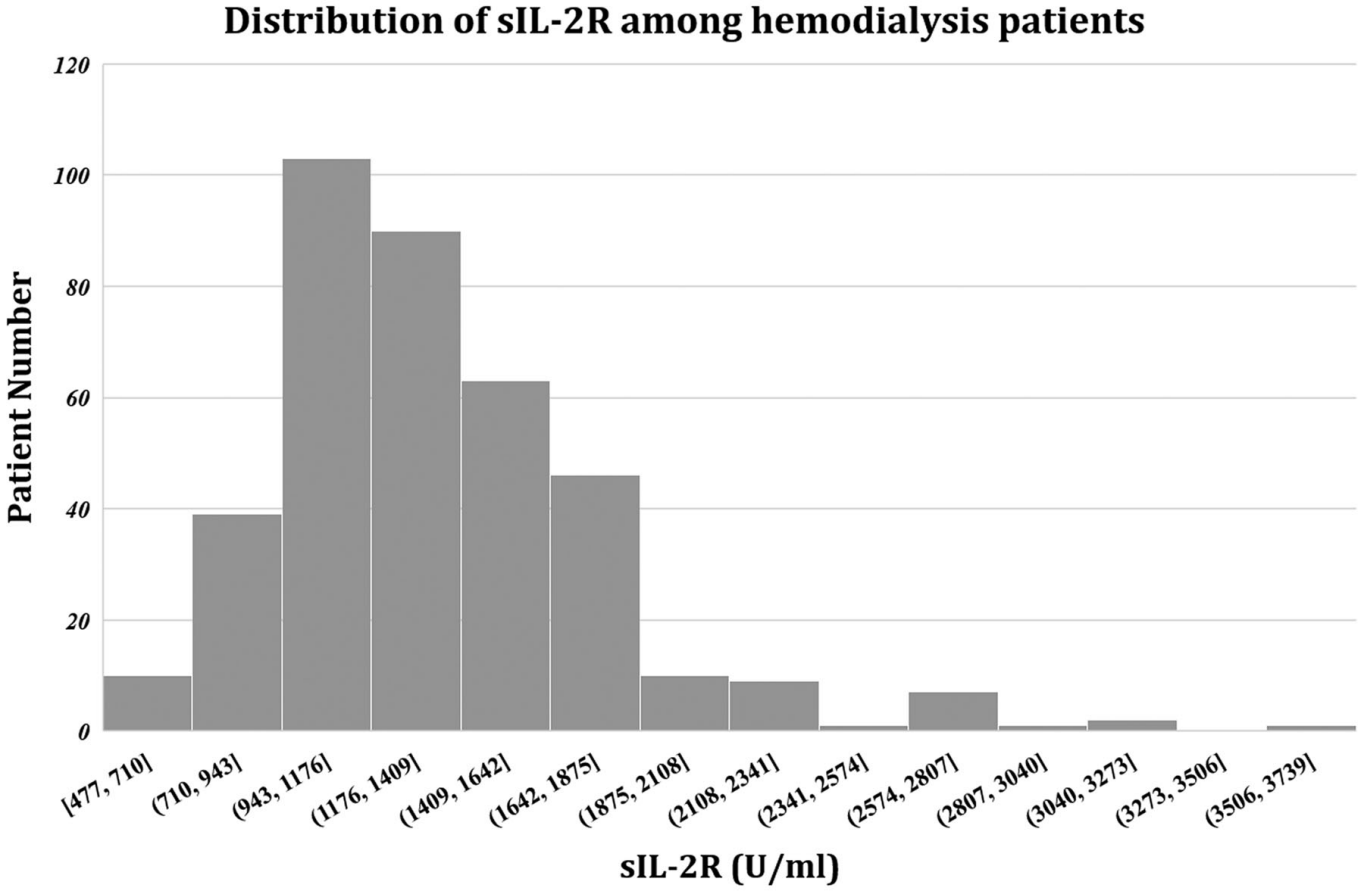
Distribution of serum sIL-2R levels in MHD patients. sIL-2R levels ranged from 477 to 3597 U/ml, with a median concentration of 1268 U/mL (IQR, 1054–1546.75 U/mL).

sIL-2R levels ranged from 477 to 3597 U/mL, with a median concentration of 1268 U/mL (IQR, 1054–1546.75 U/mL). A total of 372 (97.38%) patients exhibited sIL-2R levels higher than the upper limit of the normal range (Figure 1).

### Characteristics of patient in the low- and high-sIL-2R groups

Patients were grouped into the high sIL-2R group if their sIL-2R levels were >2-fold the upper limit of normal, and the low sIL-2R group otherwise. A comparison of patient characteristics between two groups is presented in Table 1.

**Table 1.** Comparisons between patients in high- and low-sIL-2R groups.

	Total (*N* = 382)	High sIL-2R group (*N* = 139, 36.39%)	Low sIL-2R group (*N* = 243, 63.61%)	*p* Value
Demographic characteristics				
Age, years	61 (52, 69)	63 (56, 72)	59 (50, 68)	0.0034
Male, *N* (%)	234 (61.26%)	85 (61.15%)	149 (61.32%)	0.974
BMI, kg/m^2^	22.48 (20.70, 24.67)	22.84 (21.45, 25.59)	22.31 (20.57, 24.60)	0.1634
Diabetes, *N* (%)	109 (28.53%)	52 (37.41%)	57 (23.46%)	0.004
Hepatitis-seropositive status, *N* (%)	33 (8.64%)	18 (12.95%)	15 (6.17%)	0.023
History of cancer, *N* (%)	46 (12.04%)	18 (12.95%)	28 (11.52%)	0.680
Dialysis three times weekly, *N* (%)	356 (93.19%)	129 (92.09%)	228 (93.83%)	0.516
Dialysis vintage, years	4.59 (2.27, 7.31)	4.33 (2.05, 7.81)	4.7 (2.38, 7.13)	0.6185
AVF access use, *N* (%)	307 (80.37%)	111 (79.86%)	196 (80.66%)	0.849
spKt/V	1.44 (1.26, 1.63)	1.38 (1.23, 1.61)	1.45 (1.28, 1.63)	0.1465
ESA dosage (units/week)	10,000 (5000, 15,000)	10,000 (10,000, 15,000)	10,000 (5000, 15,000)	0.0079
Cause of ESRD				
Primary glomerulonephritis, *N* (%)	200 (52.36%)	64 (46.04%)	136 (55.97%)	0.062
Diabetes mellitus, *N* (%)	73 (19.11%)	38 (27.34%)	35 (14.40%)	0.002
Hypertension, *N* (%)	38 (9.95%)	12 (8.63%)	26 (10.70%)	0.516
Polycystic kidney disease, *N* (%)	36 (9.42%)	14 (10.07%)	22 (9.05%)	0.743
Obstructive uropathy, *N* (%)	19 (4.97%)	4 (2.88%)	15 (6.17%)	0.221
Tubulointerstitial nephritis, *N* (%)	7 (1.83%)	3 (2.16%)	4 (1.65%)	0.708
Others, *N* (%)	9 (2.36%)	4 (2.88%)	5 (2.06%)	0.729
Laboratory characteristics				
sIL-2R, U/mL	1268 (1054, 1546.75)	1660 (1513, 1853)	1100 (965, 1251)	<0.0001
Neutrophil counts, 1 × 10^9^/L	4.2 (3.3, 5.1)	4.4 (3.3, 5.6)	4.1 (3.3, 4.9)	0.0721
Lymphocyte counts, 1 × 10^9^/L	1.2 (1.0, 1.6)	1.2 (1.0, 1.6)	1.3 (1.0, 1.6)	0.2779
Monocyte counts, 1 × 10^9^/L	0.51 (0.39, 0.66)	0.57 (0.42, 0.72)	0.47 (0.37, 0.61)	0.0009
Hemoglobin, g/L	114 (104, 122.25)	111 (100, 120)	117 (107, 124)	0.0029
hsCRP, mg/L	3.85 (1.3, 10.15)	5.7 (2.2, 11.9)	3.2 (1.1, 8.0)	0.0003
Albumin, g/L	39 (37, 41)	38 (37, 41)	40 (38, 42)	0.0010
SCr, μmol/L	1007.27 ± 274.46	917.60 ± 254.50	1058.56 ± 272.77	<0.0001
UA, μmol/L	437 (382, 496.5)	430 (382, 489)	441 (382, 503)	0.4971
Ferritin, ng/mL	245.65 (90.1, 440.13)	273.9 (98.3, 458.3)	235.2 (83.15, 428.15)	0.2401
TC, mmol/L	3.98 (3.4, 4.68)	3.84 (3.28, 4.60)	4.06 (3.47, 4.79)	0.0554
TG, mmol/L	1.47 (1.03, 2.30)	1.40 (0.88, 2.09)	1.49 (1.11, 2.42)	0.0674
LDL-C, mmol/L	2.19 (1.69, 2.72)	2.10 (1.66, 2.62)	2.22 (1.72, 2.77)	0.1176
HDL-C, mmol/L	0.97 (0.79, 1.24)	0.97 (0.78, 1.23)	0.97 (0.79, 1.25)	0.4778
Hcy, μmol/L	34.65 (26.75, 46.8)	34.8 (26.7, 44.53)	34.55 (26.65, 48.3)	0.4598
β2-MG, mg/L	38.95 (33.22, 43.08)	39.54 (34.83, 44.44)	38.36 (32.45, 42.72)	0.0172
iPTH, pg/mL	269.4(161.9, 418.65)	244.95 (136.55, 358.78)	274 (171, 452.45)	0.0906
Calcium, mmol/L	2.33 (2.17, 2.47)	2.32 (2.15, 2.47)	2.34 (2.21, 2.47)	0.3125
Phosphate, mmol/L	2.01 (1.56, 2.42)	1.87 (1.49, 2.32)	2.05 (1.62, 2.47)	0.1135
NT-proBNP, pg/mL	3690 (1716, 8494)	5651 (2212, 12242)	2944 (1535.25, 6902.75)	0.0001
Prognosis				
All-cause death, *N* (%)	103 (26.96)	50 (35.97%)	53 (21.81%)	0.003
Cardiovascular causes, *N* (%)	56 (14.66%)	22 (15.83%)	34 (13.99%)	0.626
Noncardiovascular causes, *N* (%)	47 (12.30%)	28 (20.14%)	19 (7.82%)	<0.0001

AVF: arteriovenous fistula; BMI: body-mass index; ESA: erythropoietin-stimulating agent; ESRD: end-stage renal disease; hsCRP: high-sensitivity C-reactive protein; HDL-C: high-density lipoprotein cholesterol; Hcy: homocysteine; iPTH: intact parathyroid hormone; LDL-C: low-density lipoprotein; β2-MG: beta-2-microglobulin; NT-proBNP: N-terminal pro-brain natriuretic peptide; sIL-2R: soluble IL-2 receptor; SCr: serum creatinine; TC: total cholesterol; TG: triglyceride; UA: uric acid.

Patients in the high sIL-2R group were older (*p* = 0.0034), the rate of diabetes was higher (*p* = 0.004), and ESRD was more often due to diabetes (*p* = 0.002). The percentage of hepatitis-seropositive status was also higher in this group (*p* = 0.023). Hemoglobin level was lower (*p* = 0.0029) and the ESA dosage was higher (*p* = 0.0481). In addition, monocyte count (*p* < 0.0001), hs-CRP (*p* = 0.0003), β2-MG (*p* = 0.0172), and NT-pro-BNP (*p* = 0.0001) levels were higher, while albumin (*p* = 0.0010) and SCr (*p <* 0.0001) levels were lower than in those in the low sIL-2R group.

### Factors correlated with sIL-2R levels

Relationships between sIL-2R and other clinical parameters were assessed (Table 2). Correlation analysis revealed that levels of sIL-2R were positively correlated with age, presence of diabetes, hepatitis-seropositive status, monocyte count, as well as hsCRP, β_2_-MG, and NT-proBNP levels, whereas negatively correlated with hemoglobin, albumin, SCr, and HDL-C levels. Backward stepwise multiple linear regression analysis revealed that only monocyte count (β = 0.1571, *p* = 0.01), and β_2_-MG (β = 0.2635, *p* < 0.0001), hemoglobin (β = −0.1610, *p* = 0.001), SCr (β = −0.3471, *p* < 0.0001), and HDL-C (β = −0.1091, *p* = 0.029) levels were independent factors influencing serum concentrations of sIL-2R.

**Table 2.** Correlation study on factors related to the levels of sIL-2R (IU/ml).

Variables	Simple linear regression analysis	*p* Value	Multiple linear regression analysis	*p* Value
	Correlation coefficient (*r*)		Standardized coefficient (*β*)	
Age	0.1481	0.0037		
Diabetes	0.1323	0.0062		
Hepatitis-seropositive status	0.1086	0.0339		
ESA dosage	0.1190	0.0140		
Monocyte counts	0.1652	0.0012	0.1571	0.001
hs-CRP	0.1756	0.0006		
β_2_-MG	0.2087	<0.0001	0.2635	<0.0001
NT-proBNP	0.2506	<0.0001		
Hemoglobin	−0.1515	0.0030	−0.1610	0.001
Albumin	−0.1672	0.0010		
SCr	−0.2872	<0.0001	−0.3471	<0.0001
HDL-C	−0.1015	0.0486	−0.1091	0.029

β_2_-MG: Beta-2-microglobulin; ESA: erythropoietin-stimulating agent; hsCRP: high-sensitivity C-reactive protein; HDL-C: high-density lipoprotein cholesterol; NT-proBNP: N-terminal pro brain natriuretic peptide; SCr: serum creatinine; sIL-2R: soluble IL-2 receptor.

### sIL-2R level and patient outcomes

During the follow-up period, 20 (5.24%) patients were transferred to other dialysis centers, 9 (2.36%) patients received renal transplantation, and 103 (26.96%) patients died. Of the 103 deaths during the 3-year observation period, 56 (54.37%) were the result of cardiovascular causes (e.g. cardiovascular, cerebrovascular, atherosclerotic, or other vascular diseases [9] and 47 (45.63%) were the result of non-cardiovascular causes, of which 39 (82.98%) were attributed to infection and/or cancer.

**Figure 2.**
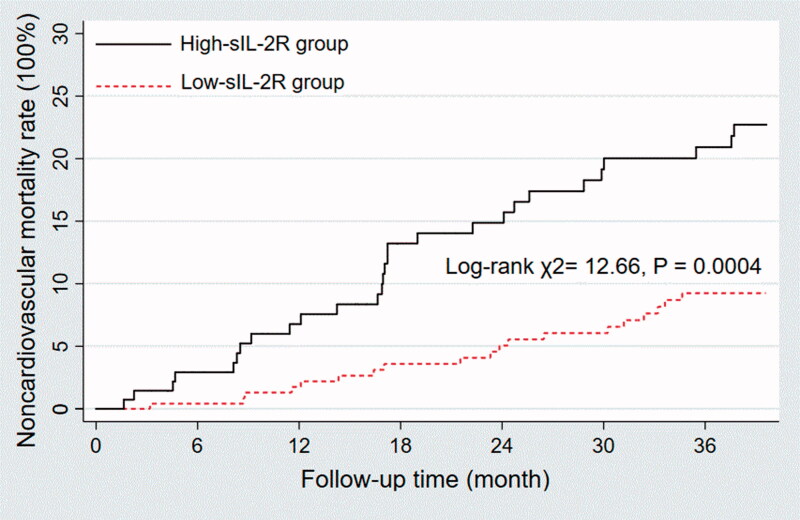
Kaplan–Meier survival curves depending on sIL-2R that was above or below the 2-fold of the upper limit of normal (710 U/ml). Patients in the high sIL-2R group exhibited a higher rate of non-cardiovascular deaths than those in the low sIL-2R group (Log rank χ2= 12.66, *p* = 0.0004).

There were more noncardiovascular deaths in the high sIL-2R group (20.14% versus 7.82%; *p* < 0.0001). Survival analysis also confirmed that patients in the high sIL-2R group exhibited a higher rate of non-cardiovascular deaths than those in the low sIL-2R group (log-rank χ^2^ = 12.66, *p* = 0.0004) (Figure 2).

Accordingly, the relationship between sIL-2R levels and noncardiovascular mortality was analyzed. In the univariate analysis, high sIL-2R (OR 2.97 [95% CI 1.59–5.56; *p* = 0.001) as well as age (per 1 year increase; OR 1.08 [95% CI 1.05–1.11; *p* < 0.0001), presence of diabetes (OR 2.05 [95% CI 1.09–3.83; *p* = 0.025), lymphocyte counts (per 1 × 10^9^/L increase, OR 0.39 [95% CI 0.19–0.83; *p* = 0.014), hs-CRP (per 1 mg/L increase, OR 1.02 [95% CI 1.01–1.03; *p* = 0.005), albumin (per 1 g/L increase, OR 0.78 [95% CI 0.70–0.86; *p* < 0.0001), SCr (per 1 mg/dL increase, OR 0.85 [95% CI 0.77–0.95; *p* = 0.003), and NT-pro-BNP (per 1 × log_10_NT-proBNP increase, OR 3.06 [95% CI 1.63–5.74; *p* = 0.001) were significant risk factors for noncardiovascular mortality (Table 3). Further multivariate stepwise regression analysis revealed that sIL-2R, whether acting as a continuous or dichotomous variable, remained significant after adjustment for other confounding risk factors (Table 4).

**Table 3.** Logistic regression analysis of risk factors related to non-cardiovascular causes mortality during the 3-year follow-up period.

Variables		Univariate	
	Unit of increase	OR (95% CI)	*p* Value
High sIL-2R group	Yes versus no	2.97 (1.59, 5.56)	0.001
Age	1 year	1.08 (1.05, 1.11)	<0.0001
Male	Yes versus no	1.26 (0.66, 2.39)	0.481
BMI	1 kg/m^2^	1.01 (0.92, 1.10)	0.909
Diabetes	Yes versus no	2.05 (1.09, 3.83)	0.025
Hepatitis-seropositive status	Yes versus no	0.98 (0.33, 2.93)	0.973
History of cancer	Yes versus no	1.91 (0.85, 4.26)	0.115
Dialysis three times weekly	Yes versus no	0.56 (0.20, 1.57)	0.271
Dialysis vintage	1 year	0.93 (0.86, 1.02)	0.107
AVF access use	Yes versus no	1.91 (0.96, 3.78)	0.065
spKt/V	1	0.99 (0.27, 3.58)	0.982
ESA dosage	5000 U/week	1.26 (0.98, 1.63)	0.075
Neutrophil counts	1 × 10^9^/L	1.07 (0.90, 1.28)	0.449
Lymphocyte counts	1 × 10^9^/L	0.39 (0.19, 0.83)	0.014
Monocyte counts	1 × 10^9^/L	2.37 (0.56, 10.00)	0.239
Hemoglobin	1 g/L	0.98 (0.97, 1.01)	0.103
hsCRP	1 mg/L	1.02 (1.01, 1.03)	0.005
Albumin	1 g/L	0.78 (0.70, 0.86)	<0.0001
SCr	1 mg/dL	0.85 (0.77, 0.95)	0.003
UA	1 mg/dL	0.89 (0.73, 1.10)	0.296
Ferritin	100 ng/mL	1.05 (0.94, 1.17)	0.400
TC	1 mmol/L	0.93 (0.69, 1.25)	0.634
TG	1 mmol/L	0.97 (0.77, 1.22)	0.783
LDL-C	1 mmol/L	0.90 (0.62, 1.30)	0.565
HDL-C	1 mmol/L	1.69 (0.72, 3.96)	0.230
Hcy	1 μmol/L	0.99 (0.97, 1.01)	0.100
β2-MG	1 mg/L	1.03 (0.99, 1.07)	0.169
iPTH	1 pg/mL	0.99 (0.88, 1.11)	0.856
Calcium	1 mmol/L	0.50 (0.19, 1.32)	0.159
Phosphate	1 mmol/L	0.72 (0.44, 1.19)	0.203
Log_10_NT-proBNP	1	3.06 (1.63, 5.74)	0.001

AVF: arteriovenous fistula; BMI: body-mass index; β2-MG: beta-2-microglobulin; CI: confidence interval; sIL-2R: soluble IL-2 receptor; ESA: erythropoietin-stimulating agent; HDL-C: high-density lipoprotein cholesterol; Hcy: homocysteine; hsCRP: high-sensitivity C-reactive protein; iPTH: intact parathyroid hormone; LDL-C: low-density lipoprotein; NT-proBNP: N-terminal pro brain natriuretic peptide; OR: odds ratio; SCr: serum creatinine; TC: total cholesterol; TG: triglyceride; UA: uric acid.

**Table 4.** Multivariate Logistic regression analysis of noncardiovascular causes mortality (sIL-2R entered as a continuous or dichotomous variable).

Variables	OR (95% CI)	*p* Value
sIL-2R, per 1 U/mL increase (continuous variable)		
Unjusted	1.001104 (1.000503, 1.001706)	<0.0001
Model 1	1.00077 (1.000121, 1.001419)	0.020
Model 2	1.000775 (1.000126, 1.001424)	0.019
sIL-2R, high versus low (dichotomous variable)		
Unjusted	2.97 (1.59, 5.56)	0.001
Model 1	2.28 (1.17, 4.48)	0.016
Model 2	2.30 (1.17, 4.50)	0.015

Model 1: adjusted for factors related with sIL-2R, including age, the presence of diabetes, hepatitis-seropositive status, ESA dosage, monocyte counts, hemoglobin, hs-CRP, albumin, SCr, HDL-C, β2-MG and NT-pro-BNP. Model 2: adjusted for factors with *p* < 0.05 in the univariate analysis, including age, the presence of diabetes, lymphocyte counts, hs-CRP, albumin, SCr, and NT-pro-BNP. CI: confidence interval; OR: odds ratio; sIL-2R: soluble IL-2 receptor.

**Figure 3.**
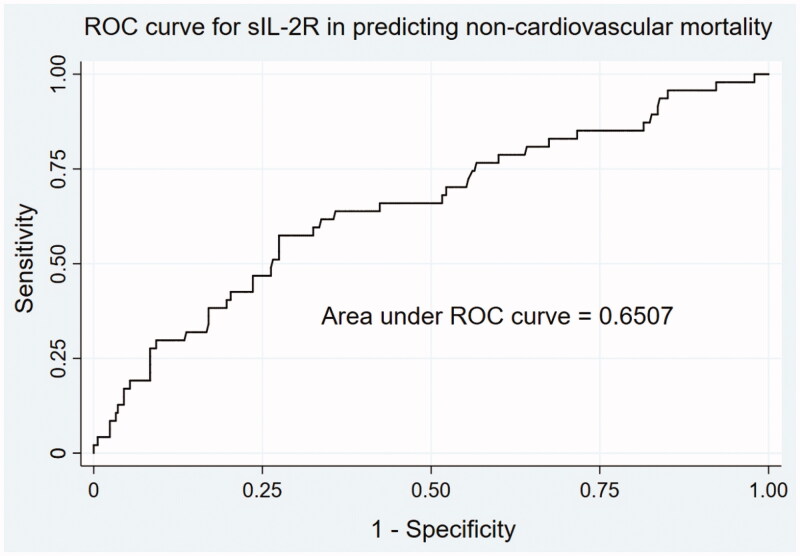
ROC curve for sIL-2R in predicting noncardiovascular mortality in MHD patients. The AUC was 0.6507 (95% CI 0.5622–0.7393). The optimal cutoff value in the ROC analysis was 1476U/ml, with a sensitivity of 57.45% and specificity of 72.54%.

ROC analysis was used to assess the performance of sIL-2R in predicting non-cardiovascular causes of mortality (Figure 3). The AUC was 0.6507 (95% CI 0.5622–0.7393). The optimal cutoff value in the ROC analysis was 1476 U/mL, which was nearly 2-fold the upper limit of normal value, with a sensitivity of 57.45% and specificity of 72.54%.

## Discussion

sIL-2R is released by activated immunocytes and involved in cell-mediated immunity during physiological and several other pathological processes. Elevated levels of sIL-2R have been observed in patients with immune diseases, inflammation, infections, lymphoproliferative disorders, cancers, or transplant rejections, and its concentration is associated with the presentation and activity of disease(s) [8]. MHD patients also exhibited an increased level of sIL-2R compared to the general population. In this study, >95% of subjects exhibited levels higher than the normal reference value (710 U/mL), with a median concentration of 1268 U/mL (IQR, 1054–1546.75 U/mL). Because sIL-2R is excreted and catabolized by the kidneys [10], it appears reasonable that patients with decreased renal function exhibit increased levels of sIL-2R. However, decreased clearance due to renal impairment is not the only determinant [11]. The authors of two previous studies noted that although uremic patients had a higher level of sIL-2R compared with healthy subjects, the highest level was observed in those undergoing MHD [6,12]. This further increase—by nearly 2-fold—was attributed to the effect of HD therapy [6]. In uremia patients undergoing MHD, a high proportion of peripheral blood mononuclear cells with phenotypic and functional signs of pre-activation was observed [3,6,12]. Spontaneous expression of IL-2R in this pre-activated state causes elevated secretion of sIL-2R in the circulation. However, in non-dialysis patients, spontaneous activation of mononuclear cells is absent [6]. Other studies have argued that pre-activation of mononuclear cells was presented in the uremic state, irrespective of whether undergoing dialysis, but HD per se exacerbated the intensity of this activation [12].

Mechanisms of immune cell activation in the setting of chronic HD are believed to be multifactorial. HD therapy-related factors, such as dialyzer biocompatibility and dialysis fluid quality, have been reported to be associated with the enhanced release of sIL-2R [3,7,12, 13]. In patients undergoing MHD, the level of sIL-2R can be increased dramatically by nearly 3–30 fold compared with that in healthy individuals [6,7,11,12,14–16]. In view of this considerable variations, the correlations between sIL-2R and patient-related factors were also examined. According to previous studies, factors including hemoglobin concentrations [15], degree of uremia [15], vitamin D levels [17], and zinc supplementation [16] were associated with increased production of sIL-2R in patients undergoing MHD. Our results suggested that the concentration of sIL-2R was significantly correlated with the level of monocytes, β_2_-MG, hemoglobin, SCr, and HDL-C, all of which appear to be involved in the inflammatory process. Monocytes play a key role in inflammation and contribute to an increased production of proinflammatory cytokines in dialysis patients [18]. β_2_-MG, a well-known marker of middle-molecular-weight uremic toxin, is an important cause of inflammation, and may stimulate excessive production of cytokines in uremic patients [19]. Anemia is another important element that is associated with the levels of several proinflammatory cytokines [20]. SCr, as a muscle mass surrogate in dialysis patients, also has a complex association with inflammation [21,22]. As for HDL-C, research involving non-ESRD patients revealed that it was inversely correlated with the level of sIL-2R because it inhibited the activation of inflammatory cells and reduced the expression of chemokines [23]. In addition to the above biomarkers, combined diseases associated with inflammation, such as diabetes or hepatitis infection, appeared to predispose patients to elevated levels of sIL-2R [7,24]. We also assessed several HD-related factors in the present study. However, both vascular access and dialysis frequency had no significant impact on sIL-2R production. We did not evaluate the influence of dialyzer biocompatibility or dialysis fluid quality because all enrolled patients were treated using uniform dialysis supplies.

As a marker of immune system activation, the presence of high concentrations of sIL-2R appears to be reasonable in uremic patients undergoing MHD. In addition to its role as a biomarker, sIL-2R *per se* has functional effects on immune responses because it interacts with IL-2 and, thereafter, modifies IL-2 signaling [25]. Substantial evidence has confirmed the biological role of sIL-2R in immune regulation and its relationship with clinical outcomes in many diseases, including lymphoma, solid cancer, and autoimmune diseases [8,26–28]. Although the increasing concentration of sIL-2R in the serum of MHD patients has been well documented, its pathophysiological consequences in this population have not been definitively clarified. In this study, we first evaluated whether the phenomenon of elevated sIL-2R levels in MHD patients could provide some information about outcomes. The results demonstrated that elevated levels of sIL-2R were significantly correlated with poor overall survival, although it was not a powerful predictor. Analysis of specific causes of death revealed that sIL-2R had no ability to predict cardiovascular-related mortality, which accounted for approximately one-half of all deaths. However, sIL-2R may be beneficial in predicting non-cardiovascular-related mortality.

It is well known that immunoactivation and immunosuppression coexist in ESRD patients [29–31]. Immune suppression and immune activation in the uremic environment have been reported to be closely linked to several complications of ESRD. Immunoactivation contributes to inflammation and accelerated tissue degeneration, thereby increasing the risk for cardiovascular disease (CVD), while immunosuppression contributes to susceptibility to infection, high risk for cancer, and poor response to vaccination [32]. Currently, infection and cancer are the leading causes of death in MHD patients following CVD [18,33–36]. Our results revealed that those with elevated levels of sIL-2R significantly increased the incidence of noncardiovascular deaths, which were mainly attributed to infection and cancer; and its relationship with noncardiovascular mortality was independent of other confounding risk factors, including age, presence of diabetes, hepatitis-seropositive status, ESA dosage, monocyte counts, hemoglobin, hs-CRP, albumin, SCr, HDL-C, β2-MG, and NT-pro-BNP. In terms of this, although sIL-2R was produced by activated immune cells, its increase appeared to contribute to immunosuppression in MHD patients, which may be related to the immunological effect of sIL-2R by suppressing IL-2 signaling and activating regulatory T cells to promote immunosuppression and tolerance [37]. Further studies are needed to determine the exact role of sIL-2R in uremic patients undergoing MHD.

sIL-2R is a relatively large molecule with a molecular weight of 45 kDa [2]; and thus, it is difficult to eliminate *via* conventional HD therapy [38]. In this study, we did not examine the effect of HDF on removing sIL-2R because the frequency of HDF was the same for all enrolled patients. However, even when using high-flux HD or hemodiafiltration, the reduction ratio of sIL-2R after one session of therapy was only 30% [39]. Because the significantly increased sIL-2R in MHD patients was not only the result of impaired clearance increased, but also and more importantly, was the result of an elevated production due to the pre-activated immune cells under the conditions of uremia and dialysis, we believe that the key to lowering sIL-2R levels is the elimination of risk factors that stimulate immunocytes, not the enhanced clearance by dialysis therapy. As Donati et al. suggested, the use of more biocompatible membranes on the dialyzer could elicit less immune cell activation, and, thus less sIL-2R release [4]. Sennesael *et al.* also reported that sIL-2R levels could be decreased following the correction of anemia [15]. If the immunological effect of sIL-2R is possible, decreasing the production of sIL-2R may potentially improve sIL-2R-mediated complications in MHD patients. However, whether sIL-2R is simply a biomarker or exerts some biological effects in MHD patients, and whether the efforts to lower sIL-2R levels are worthwhile, still needed require evaluation in further studies.

The present study had several limitations, the first of which were its single-center design and relatively small sample size. Second, we did not know whether medications (including phosphate binders, vitamin D and its analogs, anti-anemia drugs, antihypertensive drugs, antihyperlipidemic agents, or antihyperglycemic agents) that MHD patients commonly use affect sIL-2R level. Third, the mechanism of the functional effect of sIL-2R in MHD patients has not been elucidated. Therefore, further multicenter investigations are warranted.
